# Transposition of the apophysis of the greater trochanter for reconstruction of the femoral head after septic hip arthritis in children: 4 children followed for more than 15 years

**DOI:** 10.3109/17453674.2010.548030

**Published:** 2011-02-10

**Authors:** Pål Benum

**Affiliations:** Department of Orthpaedics, St. Olav's University Hospital and Department of Neuroscience, Norwegian University of Science and Technology, Trondheim, Norway

## Abstract

**Background and purpose:**

Total necrosis of the femoral head after infection in children during their first months of life gives a dislocated hip with severe leg shortening. A new femoral head can be achieved with subtrochanteric osteotomy and transposition of the apophysis of the greater trochanter into the acetabulum. Previous reports have dealt with short-term results (up to 12 years). Here I present some results of this procedure 15–24 years after operation.

**Patients and methods:**

4 children aged 1–6 years with complete necrosis of the femoral head were operated on with transposition of the greater trochanter. Secondary shelf plasty was performed later in 1 child, distal femoral epiphysiodesis in another, and femoral bone lengthening in 1 child. The mean follow-up period was 19 (15–24) years.

**Results:**

A new femoral head developed in all hips. 2 of them had a spherical head with a good acetabular cover, and without any osteoarthritis except for slight reduction of cartilage height. These hips were painless, with a mobility that allowed good walking function after 16 and 24 years, respectively. In the other 2 patients, in which there was a severe acetabular dysplasia at the primary operation, the new femoral head was somewhat flattened; painful osteoarthritis led to hip replacement 15 and 21 years after trochanter arthroplasty. Even these patients had a relatively good walking function until the last couple of years before hip replacement. Maximum leg length discrepancy was 7 cm.

**Interpretation:**

Trochanter arthroplasty with subtrochanteric osteotomy in total femoral head necrosis after septic arthritis in children may give satisfactory long-term results provided adequate acetabular cover is obtained. Although the method cannot provide a normal hip, it can contribute to less length discrepancy, less pain, improved gait, and more favorable conditions for later hip replacement.

Acute septic arthritis of the hip in children during their first months of life occasionally leads to total necrosis of the femoral head. This results in dislocation of the joint and severe limb shortening. A 6-year-old girl suffering from this condition was admitted to our department in 1985. Bearing in mind that apophyseal cartilage has the potential to develop into a kind of joint cartilage when transplanted to a joint (Benum 1974), I transposed the apophysis of the greater trochanter into the acetabulum in an attempt to reconstruct the femoral head. Encouraged by the short-term result after this operation, and by medium-term results reported after similar operations (Hunka et al. 1982, Dal Monte et al. 1984), I later used this method in 3 other children. I now present the method and the long-term results after this procedure.

## Patients and methods

**Figure 1.**
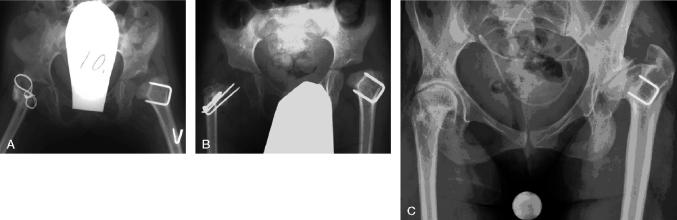
Patient no. 1. A. Preoperatively, showing no femoral head on the right side and the femur positioned considerably more proximally than normal. A valgization osteotomy had been performed previously. A valgization osteotomy had also been performed in the left hip. B. Postoperatively, following transposition of the greater trochanter into the acetabulum on the right side. The subtrochanteric osteotomy was fixated with a Steinman pin. C. 24 years postoperatively, showing a spherical femoral head well covered by a congruent acetabular roof. The cartilage thickness seems adequate. Note the steep femoral neck and the low medial femoral head offset. Severe osteoarthritic changes can be seen in the left hip.

2 girls and 2 boys were operated on (Table 1). They had all developed necrosis of the femoral head due to hip infection following sepsis during their first months of life. In 2 of the children, the sepsis was due to infection with *Staphylococcus aureus* during intravenous treatment in the first week after birth. One of the children had been treated with antibiotics because of a suspected joint infection at the age of two months. The reason for infection was unknown in 1 child, who was born in India. One of the children (no. 1), who suffered from congenital hydrocephalus, had previously been operated with a valgus osteotomy of the actual right hip since a very small remnant of a cartilaginous head without any continuity to a very short femoral neck had been found at arthrotomy. The opposite (left) hip of this patient had also been treated with a valgization osteotomy (Figure 1). It is uncertain whether the varus deformity of the left hip was due to infection of the joint or congenital dysplasia.

**Table 1.** Age at primary disease, type of infection, age at trochanter arthroplasty, and secondary operations

Patient	Age at primary disease	Reason for sepsis	Bacteriology	Affected joints	Age at arthroplasty (years)	Secondary operation
1, F	3 weeks	Intravenous infusion	*S. aureus*	Both hips	6	None
2, F	10 days	Intravenous infusion	*S. aureus*	Both hips, left ankle	1	Epiphysiodesis
3, M	6 months	Unknown	Unknown	Right hip	3	Lengthening osteotomy
4, M	2 months	Unknown	Unknown	Right hip	6	Shelf operation

**Figure 2.**
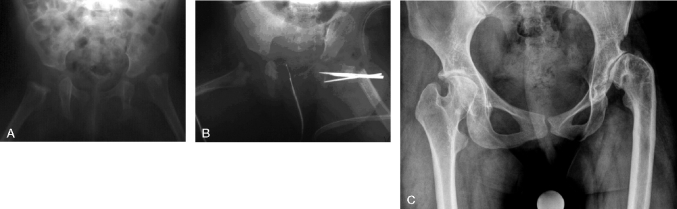
Patient no 2. A. Preoperatively, showing a luxated hip without any signs of a femoral neck and femoral head in the left hip. B. After trochanter arthroplasty and subtrochanteric osteotomy fixated with Steinman pins. Note severe acetabular dysplasia. C. 21 years after trochanter arthroplasty. The femoral head is flattended and poorly covered by the acetabulum. Osteoarthritic changes are present.

**Figure 3.**
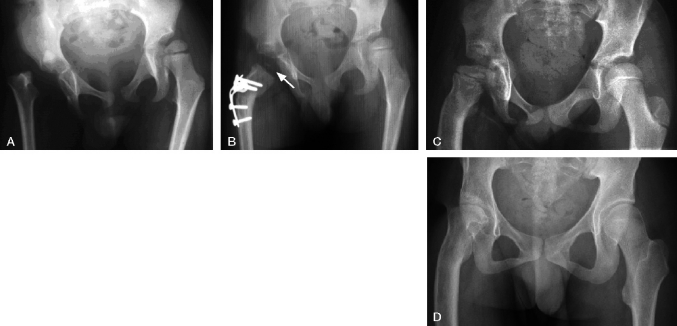
Patient no 3. A. Preoperatively, showing a totally luxated hip. B. 18 months after arthroplasty, the osteotomy had been fixated with a plate. The arrow points to a tiny ossification center in the new femoral head. C. 6 years after arthroplasty. Note the growth plate below the transposed greater trochanter. D. 6 years postoperatively. The new head is nearly spherical and well covered by a congruent acetabular roof.

**Figure 4.**
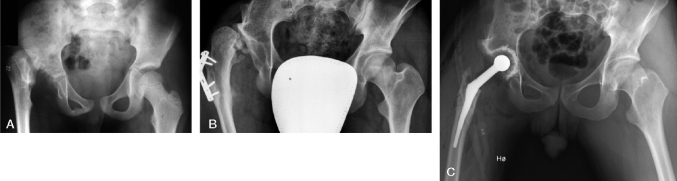
Patient no 4. A. Preoperatively, showing a luxated hip without any signs of a femoral head. Note the severe dysplasia of the acetabulum. B. 15 years after operation, showing that the new femoral head, which is nearly spherical, is poorly covered by a neoacetabulum above a severely dysplastic acetabulum. Osteoarthritis has developed. C. After insertion of a total hip prosthesis.

Age at arthroplasty ranged from 1 to 6 years. At the time of operation, all the children had a proximally dislocated femur and shortening of the limb (Figures 1–4). An anterior approach was used. Total necrosis of the femoral head was found at the operation in all of the children. In child no. 1, in whom a small remnant of a cartilaginous femoral head had been found at the previous operation, there was still no continuity to the remnants of the femoral neck. After removal of fibrous tissue from the acetabulum, the insertion of the gluteal tendons and the upper part of the insertion of the external rotator tendons were released. Then a subtrochanteric osteotomy was performed. The apophysis was slightly trimmed to fit into the acetabulum. Following transposition of the apophysis into the acetabulum, the osteotomy was fixated with Steinmann pins, screws, or a plate—aiming at a neck-shaft angle of approximately 130 degrees. The gluteal tendons were reinserted into the lateral subtrochantric area of the femur as far distally as possible. In child no. 1, an adductor tenotomy had to be peformed to enable abduction of the hip. A spica cast was used for 3 months. In child no. 4, a shelf procedure was performed 6 months after the primary operation due to severe acetabular dysplasia. The patients have now been followed for 24, 21, 16, and 15 years, respectively.

## Results

Radiographic follow-up showed development of a new femoral head in all hips. Following appearance of a center of ossification in the transposed greater trochanter, a growth zone was visible until the end of the growth period, as shown in Figure 3. In 2 of the hips a nearly spherical head developed, covered by a well-developed acetabulum (Figures 1 and 3). At the final follow-up 24 and 16 years postoperatively, when the patients were 30 and 19 years old, radiographs showed a slight reduction in cartilage height. These hips were painless, with a mobility that allowed good walking function. The other 2 hips, where a good cover for the new femoral head had not been achieved, a dysplastic joint developed (Figures 2 and 4). These hips were also painless, and the operation gave an improved gait until secondary osteoarthritis developed and gave indication for hip replacement, 21 and 15 years after primary operation when the patients were 22 and 21 years old, respectively.

During growth, a coxa valga with a reduced medial femoral head offset developed in all hips and some degree of limping was seen in all patients. Leg length discrepancy was treated with distal femoral epiphysiodesis in 1 child (no. 2), and lengthening osteotomy of the femur in another (no. 3) (Table 1). At final follow-up, the leg length discrepancies were less than 3 cm, except in 1 patient. The average total range of motion was 120 (105–135) degrees (Table 2).

**Table 2.** Findings at the final follow-up examination

Patient	Obs. period (years)	Radiographic findings			Pain **^c^**	Clinical findings at final follow-up							Limp **^d^**
		Evaluation of the joint		Leg length difference **^b^**		Range of motion							
		shape	OA **^a^**	(cm)		Ext.	Flex.	Abd.	Add.	Int. rot.	Ext. rot.	Total	
1, F	24	Spherical	no	0 (+2.0) **^e^**	None	–5	75	10	10	10	5	105	Slight
2, F	21	Dysplastic	yes	–1.3 (–2.4) ****	Moderate	0	80	15	20	10	10	135	Marked
3, M	16	Spherical	no	–2.0	None	0	90	20	10	10	5	135	Slight
4, M	15	Dysplastic	yes	–5.0 (–7.0) ****	Moderate	0	90	15	0	0	0	105	Marked

**^a^** No OA means no osteoarthritic changes except slight reduction in cartilage height.

**^b^** Values in parentheses indicate leg length difference including the difference due to subluxation.

**^c^** Moderate pain indicates pain during activity.

**^d^** Slight limp indicates limp that does not significantly influence the walking capacity, in contrast to marked limp.

**^e^** In patient no. 1, the hip on the opposite side was subluxated.

## Discussion

As early as in 1874, severe loss of bone from the femoral head following septic arthritis of the hip was described by Thomas Smith at St. Bartholomew's Hospital in London (Smith 1874). He showed by postmortem studies that the head and neck had been totally destroyed in some cases. In clinical practice, the differential diagnosis between necrosis of the femoral head due to septic arthritis and severe dysplasia of the hip may sometimes be difficult. In my series, the operative findings strongly indicated that joint infection was the reason for femoral head necrosis even in the 2 children without information about bacterial growth.

An attempt to stabilize the hip in a child with total necrosis of the femoral head was reported by L'Episcopo (1936). He split the proximal end of the remaining femur and bent the medial portion into the acetabulum. The Colonna procedure, as used in non-unions of the femoral neck (Colonna 1935), was also occasionally used in necrosis of the femoral head. However, according to the reports by Hallel and Salvati (1977) and Freeland et al. (1980), the hips tended to end up in a dislocated or subluxated position when this method was used in femoral head necrosis following septic arthritis. Stiffness and ankylosis were also mentioned as problems with this procedure.

Primary subtrochanteric osteotomy combined with transposition of the trochanteric apophysis was first described by Weissman (1967). In his case, the procedure resulted in an ankylosed hip. Furthermore, Stetson et al. (1968) reported one case operated with the same procedure. There was practically no motion in this hip 11 years after operation. Hunka et al. (1982) reported satisfactory results in 3 of 5 children 8–12 years after trochanteric arthroplasty with primary osteotomy. Monte et al. (1984) described good results in 6 of 16 children at an average of 4 years after secondary osteotomy. Finally, Dobbs et al. (2003) presented results after 5 such operations. 1 hip that was observed for 16 years had nearly autofused, whereas one hip observed for 18 years had a flattened femoral head and moderate osteoarthritic changes.

In my study of 4 hips, satisfactory results with development of a spherical femoral head well contained within a nearly concentric and well-located acetabulum were found in 2 hips, 24 and 16 years after surgery. In both of these cases, the acetabulum gave a nearly normal cover of the femoral head after the operation. In the 2 cases where substantial osteoarthritic changes developed, a severely dysplastic acetabulum gave insufficient support to the reconstructed femoral head. This demonstrates that satisfactory long-term results depend on adequate acetabular cover after operation. It should be noticed, however, that the method may give reasonably good function of the hip during childhood and adolescence, and to some degree prevent development of severe shortening of the limb, even if ideal cover of the femoral head is not obtained.

Some tendency to limp was seen in all patients, as in other studies of patients operated with this procedure. This is probably partly caused by the reduced lever arm of the gluteal muscles due to the development of coxa valga. The growth plate of the greater trochanter only contributes to increased length of the new femoral neck and femoral shaft after arthroplasty, and not to increased width of the proximal femur at the level of the insertion of the gluteal muscles. Although the growth plate contributes to increased length of the femur, this contribution is too small to fully compensate for the loss of the original proximal femoral growth plate. Thus, secondary operative procedures to avoid unacceptable leg-length discrepancies might still be indicated after this type of arthroplasty, as done in 2 of my patients. However, the leg length discrepancies will usually be less in children in whom trochanter arthroplasty is performed than in untreated patients, since the method prevents severe proximal migration of the femur.

From a biological point of view, it is interesting that an apophysis has the potential to develop into an epiphyseal-like structure when traction is replaced by pressure and friction. The osseous nucleus grows and is remodeled into a spherical joint-end which adapts to the opposite joint surface. Under normal conditions, all the cartilage of an apophysis is completely ossified at the end of the growth period. After transposition to the joint, this process seems to be modified leaving the most superficial layers of the new articulating surface unossified, like what was seen when apophyseal cartilage was transplanted to joint defects (Benum 1974).

There is no agreement about the most suitable age for doing a trochanter arthroplasty. Probably the apophysis has the greatest potential for remodeling before the ossification has become too advanced. On the other hand, it seems likely that the remodeling process is greatly influenced by the loading and motion that takes place during gait. Hence, the best results will probably be obtained if the child has already passed the first learning period of walking. In my child patients, all operations were performed before the age of 7 years. As in other studies, however, the number of hips was too small in this study to be able to draw any firm conclusions about the ideal age for this type of operation.

In summary, previous studies have shown some satisfactory results up to 12 years after subtrochanteric osteotomy and transposition of the apophysis of the greater trochanter into the acetabulum in children with total femoral head necrosis due to septic arthritis. My findings indicate that satisfactory results may even last for a considerably longer period. A nearly perfect acetabular cover of the transposed apophysis is a prerequisite for a long-lasting favorable result. However, the procedure might be beneficial even if a perfect cover is not achieved. In such a case, the operation might reduce the tendency to shortening of the limb and give a reasonable function until osteoarthritis develops. Furthermore, it appears likely that a total hip replacement might give a better result in such a hip compared to an untreated hip with total dislocation and severe shortening
